# Supplementary material for: Breast and Colorectal Cancer Screening Utilization after Hurricane María and the COVID-19 Pandemic in Puerto Rico
Source: Int J Environ Res Public Health. 2023 Oct 1;20(19):6870. doi: 10.3390/ijerph20196870 (PMC10572647; doi:10.3390/ijerph20196870)
Supplement: Supplementary file 1 [file ijerph-20-06870-s001.zip › ijerph-2562784-supplementary.pdf]

Supplementary Materials

Table S1. CPT & HCPCS codes used in this study.

Codes to Identify Cancer Screening	CPT & HCPCS Codes	Description
Colorectal cancer screening	82270	fecal occult blood test (FOBT)
	81528	quantitative real-time target and signal amplification of 10 DNA markers
	G0328	Fecal Immunochemical Testing (FIT)
	G0104	flexible sigmoidoscopy
	G0105	colonoscopy on individual at high risk
	G0121	colonoscopy on an individual not meeting criteria for high risk
	G0464	stool-based DNA and fecal occult hemoglobin
Breast cancer screening	77057, 77067	screening mammography, bilateral (2-view study of each breast)
	G0202	screening mammography, bilateral (2-view study of each breast), including computer-aided detection Computer-Aided Detection (CAD) when performed
	77063	screening digital breast tomosynthesis

Table S2. Estimated Coefficients for the Poisson model to fit the number of claims for colorectal cancer screening controlling for year, month, age-group, sex, and number of beneficiaries.

Parameter	Estimated Coefficient (95% CI)	SE	Sig
year (ref = 2016)			
2017	0.00 (−0.07, 0.07)	0.04	0.94
2018	−0.05 (−0.12, 0.02)	0.04	0.19
2019	0.01 (−0.06, 0.08)	0.04	0.71
2020	−0.16 (−0.23, −0.09)	0.04	0.00
2021	−0.18 (−0.25, −0.11)	0.04	0.00
Month (ref = Jan)			
Feb	0.29 (0.23, 0.35)	0.03	0.00
Mar	0.31 (0.25, 0.38)	0.03	0.00
Apr	0.40 (0.33, 0.46)	0.03	0.00
Jun	0.25 (0.18, 0.31)	0.03	0.00
May	0.25 (0.19, 0.31)	0.03	0.00
Jul	−0.01 (−0.07, 0.06)	0.04	0.87
Aug	0.19 (0.12, 0.25)	0.03	0.00
Sep	0.10 (0.01, 0.15)	0.03	0.01
Oct	0.08 (−0.24, −0.10)	0.03	0.02
Nov	−0.17 (−0.46, −0.31)	0.04	0.00
Dec	−0.39 (0.03, 0.16)	0.04	0.00
Age groups (ref = 40–49)			
50–59	0.91 (0.89, 0.92)	0.01	0.00
60–64	1.03 (1.02, 1.05)	0.01	0.00
65–69	0.94 (0.91, 0.96)	0.01	0.00
70–74	0.94 (0.91, 0.97)	0.02	0.00
Gender (ref = Female)			
Male	−0.23 (−0.24, −0.22)	0.01	0.00
Interaction (Year * Month)			
2017 * Feb	−0.06 (−0.15, 0.03)	0.05	0.00
2018 * Feb	−0.08 (−0.17, 0.01)	0.05	0.05
2019 * Feb	−0.11 (−0.21, −0.02)	0.05	0.00
2020 * Feb	0.07 (−0.02, 0.17)	0.05	0.00
2021 * Feb	−0.02 (−0.11, 0.08)	0.05	0.00
2017 * Mar	0.00 (−0.09, 0.09)	0.05	0.00
2018 * Mar	−0.08 (−0.17, 0.02)	0.05	0.15
2019 * Mar	−0.10 (−0.19, −0.01)	0.05	0.00
2020 * Mar	−0.52 (−0.62, −0.42)	0.05	0.00
2021 * Mar	0.10 (0.01, 0.19)	0.05	0.00

2017 * Apr	−0.29 (−0.38, −0.19)	0.05	0.00
2018 * Apr	−0.09 (−0.18, 0.00)	0.05	0.00
2019 * Apr	−0.37 (−0.46, −0.28)	0.05	0.00
2020 * Apr	−2.13 (−2.28, −1.98)	0.08	0.13
2021 * Apr	−0.27 (−0.36, −0.17)	0.05	0.00
2017 * May	−0.15 (−0.25, −0.06)	0.05	0.18
2018 * May	0.02 (−0.07, 0.11)	0.05	0.09
2019 * May	−0.27 (−0.37, −0.18)	0.05	0.01
2020 * May	−1.03 (−1.14, −0.91)	0.06	0.13
2021 * May	−0.15 (−0.24, −0.05)	0.05	0.70
2017 * Jun	−0.23 (−0.33, −0.14)	0.05	0.00
2018 * Jun	−0.09 (−0.18, 0.00)	0.05	0.80
2019 * Jun	−0.41 (−0.51, −0.31)	0.05	0.00
2020 * Jun	−0.48 (−0.58, −0.38)	0.05	0.19
2021 * Jun	−0.18 (−0.28, −0.09)	0.05	0.18
2017 * Jul	−0.17 (−0.27, −0.07)	0.05	0.00
2018 * Jul	0.01 (−0.09, 0.11)	0.05	0.06
2019 * Jul	−0.28 (−0.38, −0.18)	0.05	0.00
2020 * Jul	−0.07 (−0.17, 0.03)	0.05	0.00
2021 * Jul	−0.07 (−0.17, 0.03)	0.05	0.00
2017 * Aug	−0.20 (−0.3, −0.11)	0.05	0.99
2018 * Aug	−0.07 (−0.16, 0.02)	0.05	0.11
2019 * Aug	−0.40 (−0.50, −0.30)	0.05	0.03
2020 * Aug	−0.26 (−0.36, −0.16)	0.05	0.00
2021 * Aug	−0.30 (−0.40, −0.20)	0.05	0.03
2017 * Sep	−1.07 (−1.18, −0.95)	0.06	0.00
2018 * Sep	−0.13 (−0.23, −0.04)	0.05	0.66
2019 * Sep	−0.44 (−0.54, −0.34)	0.05	0.00
2020 * Sep	−0.15 (−0.25, −0.04)	0.05	0.00
2021 * Sep	−0.24 (−0.35, −0.14)	0.05	0.00
2017 * Oct	−1.49 (−1.62, −1.36)	0.07	0.00
2018 * Oct	−0.12 (−0.21, −0.02)	0.05	0.00
2019 * Oct	−0.30 (−0.40, −0.20)	0.05	0.00
2020 * Oct	−0.05 (−0.15, 0.05)	0.05	0.00
2021 * Oct	−0.27 (−0.37, −0.17)	0.05	0.00
2017 * Nov	−0.52 (−0.63, −0.41)	0.06	0.00
2018 * Nov	−0.42 (−0.53, −0.31)	0.06	0.02
2019 * Nov	−0.31 (−0.42, −0.20)	0.05	0.00
2020 * Nov	−0.20 (−0.31, −0.09)	0.06	0.30
2021 * Nov	−0.19 (−0.29, −0.08)	0.06	0.00
2017 * Dec	−0.43 (−0.55, −0.32)	0.06	0.00
2018 * Dec	−0.44 (−0.56, −0.32)	0.06	0.01
2019 * Dec	−0.26 (−0.37, −0.15)	0.06	0.00
2020 * Dec	−0.09 (−0.2, 0.03)	0.06	0.00
2021 * Dec	−0.22 (−0.33, −0.10)	0.06	0.00

Table S3. Estimated Coefficients for the Poisson model to fit the number of claims for breast cancer screening controlling for year, month, age-group, and number of beneficiaries.

Parameter	Estimated Coefficient (95% CI)	SE	Sig
year (ref = 2016)			
2017	0.18 (0.12, 0.24)	0.03	0.00
2018	0.21 (0.15, 0.26)	0.03	0.00
2019	0.16 (0.10, 0.22)	0.03	0.00
2020	0.10 (0.04, 0.16)	0.03	0.00
2021	0.24 (0.18, 0.30)	0.03	0.00
Month (ref = Jan)			
Feb	0.37 (0.31, 0.42)	0.03	0.00
Mar	0.38 (0.32, 0.44)	0.03	0.00
Apr	0.46 (0.41, 0.52)	0.03	0.00
Jun	0.40 (0.34, 0.45)	0.03	0.00
May	0.46 (0.40, 0.52)	0.03	0.00
Jul	0.18 (0.13, 0.24)	0.03	0.00
Aug	0.47 (0.42, 0.53)	0.03	0.00
Sep	0.40 (0.34, 0.46)	0.03	0.00
Oct	0.64 (0.59, 0.69)	0.03	0.00
Nov	0.40 (0.34, 0.46)	0.03	0.00
Dec	0.23 (0.17, 0.29)	0.03	0.00
Age groups (ref = 50–59)			
60–64	0.03 (0.02, 0.04)	0.01	0.00
65–69	−0.20 (−0.22, −0.18)	0.01	0.00
70–74	−0.32 (−0.34, −0.30)	0.01	0.00
Interaction (Year * Month)			
2017 * Feb	−0.16 (−0.24, −0.08)	0.04	0.00
2018 * Feb	−0.08 (−0.15, 0.00)	0.04	0.06
2019 * Feb	−0.08 (−0.16, 0.00)	0.04	0.04
2020 * Feb	−0.02 (−0.10, 0.06)	0.04	0.67
2021 * Feb	−0.02 (−0.09, 0.06)	0.04	0.69
2017 * Mar	0.03 (−0.05, 0.11)	0.04	0.43
2018 * Mar	0.00 (−0.07, 0.08)	0.04	0.91
2019 * Mar	0.09 (0.01, 0.17)	0.04	0.02
2020 * Mar	−0.62 (−0.71, −0.54)	0.04	0.00
2021 * Mar	0.11 (0.04, 0.19)	0.04	0.00
2017 * Apr	−0.24 (−0.32, −0.17)	0.04	0.00
2018 * Apr	0.02 (−0.06, 0.09)	0.04	0.65
2019 * Apr	−0.04 (−0.12, 0.04)	0.04	0.32
2020 * Apr	−3.09 (−3.27, −2.92)	0.09	0.00
2021 * Apr	−0.27 (−0.35, −0.19)	0.04	0.00
2017 * May	−0.07 (−0.15, 0.00)	0.04	0.07
2018 * May	0.05 (−0.03, 0.13)	0.04	0.19
2019 * May	0.02 (−0.06, 0.09)	0.04	0.65
2020 * May	−1.31 (−1.41, −1.21)	0.05	0.00
2021 * May	−0.25 (−0.33, −0.18)	0.04	0.00
2017 * Jun	−0.12 (−0.20, −0.04)	0.04	0.00
2018 * Jun	−0.10 (−0.18, −0.03)	0.04	0.01
2019 * Jun	−0.13 (−0.21, −0.05)	0.04	0.00
2020 * Jun	−0.56 (−0.64, −0.47)	0.04	0.00
2021 * Jun	−0.20 (−0.28, −0.13)	0.04	0.00
2017 * Jul	−0.09 (−0.17, 0.00)	0.04	0.04
2018 * Jul	0.04 (−0.04, 0.12)	0.04	0.38
2019 * Jul	0.02 (−0.06, 0.10)	0.04	0.58
2020 * Jul	−0.06 (−0.14, 0.02)	0.04	0.16
2021 * Jul	−0.04 (−0.11, 0.04)	0.04	0.38
2017 * Aug	−0.05 (−0.13, 0.02)	0.04	0.17
2018 * Aug	−0.04 (−0.12, 0.03)	0.04	0.26

2019 * Aug	−0.13 (−0.21, −0.06)	0.04	0.00
2020 * Aug	−0.25 (−0.33, −0.17)	0.04	0.00
2021 * Aug	−0.25 (−0.32, −0.17)	0.04	0.00
2017 * Sep	−1.20 (−1.29, −1.10)	0.05	0.00
2018 * Sep	−0.20 (−0.28, −0.12)	0.04	0.00
2019 * Sep	0.01 (−0.06, 0.09)	0.04	0.71
2020 * Sep	−0.01 (−0.09, 0.07)	0.04	0.79
2021 * Sep	−0.15 (−0.23, −0.08)	0.04	0.00
2017 * Oct	−1.79 (−1.88, −1.69)	0.05	0.00
2018 * Oct	−0.29 (−0.36, −0.21)	0.04	0.00
2019 * Oct	−0.03 (−0.10, 0.05)	0.04	0.47
2020 * Oct	−0.05 (−0.12, 0.03)	0.04	0.22
2021 * Oct	−0.30 (−0.37, −0.22)	0.04	0.00
2017 * Nov	−0.87 (−0.95, −0.78)	0.04	0.00
2018 * Nov	−0.83 (−0.91, −0.74)	0.04	0.00
2019 * Nov	−0.16 (−0.24, −0.08)	0.04	0.00
2020 * Nov	−0.15 (−0.23, −0.07)	0.04	0.00
2021 * Nov	−0.16 (−0.24, −0.09)	0.04	0.00
2017 * Dec	−0.46 (−0.54, −0.38)	0.04	0.00
2018 * Dec	−0.60 (−0.69, −0.52)	0.04	0.00
2019 * Dec	−0.26 (−0.34, −0.17)	0.04	0.00
2020 * Dec	0.04 (−0.04, 0.12)	0.04	0.34
2021 * Dec	−0.19 (−0.27, −0.11)	0.04	0.00
